# Supplementary material for: Enhancing Recommendation Systems through SVD-based collaborative filtering and community detection
Source: PLoS One. 2026 Apr 13;21(4):e0346579. doi: 10.1371/journal.pone.0346579 (PMC13075704; doi:10.1371/journal.pone.0346579)
Supplement: Attachment — Submitted filename: PONE-D-25-63400_suggestions and comments.pdf [file pone.0346579.s001.pdf]

Click here to access/download;Manuscript;Enhanced Recommendation Systems- Manuscript.pdf

# Enhancing Recommendation Systems through SVD-Based Collaborative Filtering and Community Detection

T. Keerthika[1], Ignisha Rajathi G[2*], Vedhapriyavadhana Rajamani[3], Surya Santhosh Kumar[1], Karthigai Selvam[1], Aiyyappan M. R.[1], L S Thoshi Babu[1]

[1]School of Artificial Intelligence, Amrita Vishwa Vidyapeetham, Coimbatore, Tamil Nadu, India.
[2*]School of Computer Engineering, Manipal Institute of Technology Bengaluru, Manipal Academy of Higher Education, Manipal, India.
[3]School of Computing, Engineering and Physical Sciences, University of the West of Scotland, London, England, United Kingdom.

*Corresponding author(s). E-mail(s): ignisha.rajathi@manipal.edu;
Contributing authors: t_keerthika@cb.amrita.edu;
Vedhapriyavadhana.Rajamani@uws.ac.uk;
cb.en.u4aie22049@cb.students.amrita.edu;
cb.en.u4aie22053@cb.students.amrita.edu;
cb.en.u4aie22025@cb.students.amrita.edu;
cb.en.u4aie22129@cb.students.amrita.edu;

**Abstract**

The problems that are often faced by recommendation systems are low data density, scalability, and a lack of interpretability. Classical collaborative filtering algorithms based on Singular Value Decomposition (SVD) partly address such issues but, again, in extreme cases of sparsity and cold International market-entry they are ineffective. Graph Neural Networks (GNNs) will provide a possible solution to these deficiencies, but require extensive computational power and limited explanatory abilities. Due to these constraints, we have developed a hybrid system that combines SVD based collaborative and filtering and the Louvain community detection algorithm. The method utilizes SVD in dimensionality reduction as well as methods at the same time dimensionality reduce using community detections in order to allocate groups of users with similar preferences

profiles. As a result, the structure leads to better personalization, filters noise benefits of sparse information, and is more interpretable. The experimental analysis of Netflix Prize dataset has proved the effectiveness of the suggested approach as it revealed a root-mean-square error (RMSE) of 0.9966 and a mean absolute error (MAE) of 0.7968. These metrics show a high level of performance compared to the isolated SVD implementations and allow much better interpretability than GNN based approaches. The framework explains decipherable, community-based preference frameworks that lead to recommendation judgments. This architecture will be extended in the future to include the temporal modeling and the multi-domain recommendation situations.

**Keywords:** Recommendation Systems, Singular Value Decomposition, Louvain Community Detection, Collaborative Filtering

# 1 Introduction

The modern Recommendation Systems (RS) draw their base from historic recommendation methods which comprised Content-Based Filtering (CBF) and Collaborative Filtering (CF) [5, 6]. Similar preferences among users emerge when their past behaviors show overlap according to CF principles. The CF system operates within two distinct subcategories that either base recommendations on comparable user behavior or use item similarity to generate suggestions [6, 7]. The strength of CF in processing hidden item–user relationships becomes limited by its inability to address data sparsity and scalability issues [10]. The recommendation capabilities of CBF depend on item characteristics or profiles for interpretation but fall short in discovering unconscious patterns and extending knowledge outside of defined features [6].

Matrix Factorization (MF) techniques gain recognition through their powerful achievement through Singular Value Decomposition (SVD) as their key member [31]. Through SVD, latent factors are produced by breaking down the user–item interaction matrix, revealing the underlying preferences along with characteristics. Such a method decreases both data complexity and reveals previously undetected connections that standard CF solutions fail to observe [12]. The application of SVD for recommendation accuracy enhancement leads to noticeable limitations. One drawback of SVD happens when dealing with extremely sparse datasets and the system fails to detect internal relationships between users that create communities as well as when starting with new users or items [10, 13, 29].

Recent works emphasize the growing role of deep learning in collaborative filtering to overcome these exact limitations. As shown in the survey by Jothilakshmi and Bharathi [29], deep learning–based CF methods have improved the handling of cold-start and data sparsity by learning high-level feature representations.

Nowadays researchers focus on graph-based approaches to solve these gaps through which they exploit community detection among dense nodes found in user–item interaction graphs and user–user similarity networks [2, 4, 10]. The concept of communities proves essential for recommendation systems because it allows members of common

groups to receive recommendations that better match their preferences [3, 9]. Numerous community detection methods continue to evolve because they explore different algorithm functionalities for separate network applications [14, 19]. The Louvain method stands out for its high operational efficiency and its modularity-optimization capabilities, and therefore works best with extensive network systems [14, 19]. Computational efficiency stands as a weakness for Girvan–Newman, but Infomap offers an accuracy-cost balance for identifying overlapping communities in networks [6]. The algorithms provide individualized perspectives which serve as beneficial tools for RS performance optimization [3].

The Louvain method enables recommendation systems to uncover social groups that foster user-experience optimization alongside content-recommendation optimization [3, 9, 14]. The combination of collaborative filtering used by SVD with personal recommendation prediction produces an enhanced synergy which strengthens both system accuracy and diversity [7, 13]. The two methods operate in network science by enabling biological-network functional-module discovery through community detection while detecting hidden textual-data structures using SVD, supporting applications in biology and natural-language processing [6, 13]. They help decrease data-storage requirements because SVD contracts information into smaller footprints and community detection recognizes crucial data groups, supporting both minimization of storage space and efficient transmission [3, 12]. In e-commerce, SVD delivers personalized recommendations, while community detection helps segment customers for targeted marketing purposes [3, 9, 13]. Network visualization becomes simpler when SVD and community detection are combined, as dimensionality reduction and structural-pattern highlighting reveal complex relationships in an interpretable form [3, 4, 6].

Many organizations are currently turning toward Graph Neural Networks (GNNs) in their recommendation systems because GNNs can model complex relations between users and items and consequently provide very accurate recommendations [2, 17, 21]. However, GNNs also require large amounts of training data and computational power, making their use expensive and time-consuming [21, 24]. Notwithstanding such barriers, many firms still prefer GNNs as "black-box" solutions that return recommendations without exposing the internal reasoning to users [4, 22, 30].

We utilize Singular Value Decomposition (SVD) to discover latent patterns in user–item interactions and community detection techniques like Louvain to leverage the natural group structures among users [3, 13, 14]. The hybrid approach offers advantages such as lower computational needs, better interpretability, and strong performance on sparse data, without sacrificing accuracy relative to GNN-based models [2, 9, 17].

## 2 Related Work

The development of recommendation systems happens through the combination of graph-based techniques and community detection algorithms and Singular Value Decomposition (SVD) for matrix factorization. Research reveals a comprehensive review indicates graph-based recommender systems are more significant nowadays

because they extract key information from graph structures to create strong recommendations and improve their readability [5].

Scientists examine transparent graph-based recommenders by studying how graph structures enhance recommendation trust by identifying communities and representing nodes [4]. Several research documents show that graph learning methods successfully address data sparsity and cold-start challenges [10, 20].

The split procedure conducted by community detection algorithms creates distinct groups from user sets that produces superior individualized recommendation results. Extensive network applications benefit from three leading community detection methods which include Louvain, Leiden and Label Propagation [3, 6]. The Louvain method stands as the preferred approach for producing top-quality community structures making it the choice for multiple real-world applications according to [9, 19]. Recent memory-efficient development of algorithm implementations increased the scalability of these algorithms for large-scale database operations [1, 14]. The recent developments enable powerful conditions which allow community detection methods to integrate with collaborative filtering systems.

Appealing recommender systems integrate collaborative filtering and content-based techniques which have gained popularity because they deliver best accuracy and extend system diversity. Research on SVD in collaborative filtering expanded because the algorithm extracts hidden patterns from user-item data matrices [6, 7]. Hybrid recommendation systems extract strengths from dual recommendation techniques to solve sparsity limitations and overfitting issues thus improving user-specific recommendations. User preference prediction within SVD-based recommendation systems attains its robust performance thanks to implementing bias terms that apply user and item-based behavioral approaches as documented in fundamental research [12, 13].

The main reason recommendation systems rely on graph-based models reflects their ability to detect complex patterns together with user preferences. GNNs stand as a core field in graph representation which integrates node-specific information to learn recommendations for graph-based applications as per [2, 21, 24].

Research primarily investigates the techniques to model how users engage with multiple items. Xia et al. developed Multi-Behavior GNN (MB-GNN) as a model that enhances representation learning by processing different user interaction types such as clicks and likes and purchases [17]. The aggregation method across multiple channels delivers effective results when dealing with sparse data conditions that result in performance decline when using single-interaction analysis.

Recommendation systems of modern times emphasize both the transparent handling of recommendations and the capability to explain recommendation outcomes. Black-box models from the past lack any explanation regarding the reasoning behind recommendations. The adoption of hybrid models which unite latent factor models and graph-based community detection methods has seen increasing popularity in order to reverse these issues. The community detection algorithms Louvain and Label Propagation identified hidden patterns in users or items according to Gasparetti et al. [3] and Shokrzadeh et al. [9] which enhances the overall understandability of recommendations. The incorporation of graph structural details results in improved performance and boosts both user credibility and interaction with the system [4, 22].

The quantity of users and objects in the real world applications encourages the occurrence of system scalability challenges. The research by Mohammadi et al. [1] coupled with Blondel et al. [14, 19] developed memory and parallel optimization variants of community algorithms which make processing large datasets efficient while maintaining quality attributes. Community-based recommendation systems can utilize the Louvain method because it demonstrates both high scalability and efficiency in modularity computation.

SVD-based methods together with their variants remain prominent in matrix factorization because they demonstrate successful performance for extracting latent item-user relationships. The scholars at Koren et al. [12] created a bias-aware SVD model which enhanced personalization through user and item deviation handling. The practical use and success of SVD for building movie recommendation systems became apparent through Zhao et al.'s [6] current research. The techniques get increasingly adopted within hybrid models to link latent factor interpretability and GNNs structural strength [7, 25].

Research now focuses on evaluating fairness and diversity and serendipity within recommendation output results. The review study by Duricic et al. [26] established that GNN-based recommenders require beyond-accuracy metrics to achieve both good user satisfaction along with ethical and diversified content output. Recognition of such targets becomes vitally important in deploying recommendation engines to socially sensitive domains like education and hiring as well as news curation.

Multiple surveys collect the main problems and research paths for recommendation systems built with GNN technology. Research by Wu et al. [10, 21, 23] and Gao et al. [2, 24] has identified current obstacles in understanding recommendations and coping with heterogeneity and dealing with new user situations. Their research supports development of unified models that join community data elements with extra information along with time-based dependency analysis.

Presently there is a knowledge gap in literature about combining SVD and GNN-based techniques with community detection methods for producing scalable and interpretable personalized frameworks. Research has been studying these elements separately but scientists are now convinced that combining these components results in state-of-the-art hybrid models suitable for practical applications according to [3, 4, 9, 13, 17]. Such recommendation systems combine end-user effectiveness with specific details about what factors contribute to peer group and latent interest-based and graph proximity-based user decision patterns.

# 3 Methodology

## 3.1 Singular Value Decomposition (SVD)

Singular Value Decomposition (SVD) stands as a fundamental component of collaborative filtering since it offers a strong mathematical framework to extract hidden factors that explain user-item engagements. A matrix $R$ represents the user-item interaction data with each user having one row while items occupy one column and each cell shows rating or implicit recordings. Cutting-edge implementations of this matrix technique demonstrate sparsity since users predominantly access a limited number of items in

real-life systems. SVD resolves sparsity by dividing the matrix $R$ into three elements including $U$, $\Sigma$, and $V^{\mathrm{T}}$, which appear in the following mathematical expression

$$R = U \Sigma V^{\top}, \tag{1}$$

Here:

- $U$ is an $m \times k$ matrix capturing latent features of users,
- $\Sigma$ is a $k \times k$ diagonal matrix including singular values (importance weights of latent factors),
- $V^{\mathrm{T}}$ is a $k \times n$ matrix representing latent features of items.

SVD generates its main strength from discovering hidden patterns between users and items which remain hidden in original data sources. The latent features of two users with different movie rating behavior will show similar preference patterns when their vectors demonstrate close alignment. The evaluation indicates similarness when movies do not share any rating feedback because they address comparable latent factors. By predicting ratings for users and items with limited interactive history, the SVD feature is well-suited to resolving the cold-start problem. SVD uses learned latent factors from existing data to estimate rating values that enable relevant recommendations even though data is sparse and missing. The SVD method finds practical execution through FunkSVD algorithms that perform stochastic gradient descent (SGD) optimization for decomposition optimization. The regularization terms implemented in FunkSVD help prevent overfitting and achieve effective generalization for unseen data. The optimization process finds minimal squared differences between user ratings predictions and actual values by applying penalties to large latent factor values to achieve interpretation simplicity. The optimization includes the following formula:

$$\mathcal{L} = \sum_{(u,i)\in\Omega} (r_{u,i} - \hat{r}_{u,i})^2 + \lambda\big(\|p_u\|^2 + \|q_i\|^2\big) \tag{2}$$

where:

- $\Omega$ symbolizes the collection of user-item interactions,
- $r_{u,i}$ is the actual rating given by user $u$ to item $i$,
- $\hat{r}_{u,i}$ is the predicted rating, computed as:

$$\hat{r}_{u,i} = \mu + b_u + b_i + p_u^{\top} q_i, \tag{3}$$

Here:

- $\mu$ represents the global average rating,
- $b_u$ represents the user-specific bias,
- $b_i$ represents the e item-specific bias,
- $p_u$ represents the latent factor vector for user $u$,
- $q_i$ i represents the latent factor vector for item $i$,
- $p_u^{\top} q_i$ represents the dot product capturing the affinity between user $u$ and item $i$.

During the training process the system updates user and item latent factor vectors $p_u$ and $q_i$ through repeated iterations of observed interactions. The gradient descent

updates adjust the relevant latent factors whenever a user provides a rating that exceeds the prediction

$$p_u \leftarrow p_u + \alpha\big(e_{u,i} q_i - \lambda p_u\big), \tag{4}$$

$$q_i \leftarrow q_i + \alpha\big(e_{u,i} p_u - \lambda q_i\big), \tag{5}$$

where:

- $e_{u,i}$ represents the prediction error,
- $\alpha$ represents the learning rate,
- $\lambda$ represents the regularization parameter.

The prediction formula receives improvement through the implementation of performance-enhancing biases that include global average ratings alongside user-specific tendencies and item popularity elements. The predictive model adds positive bias terms to user ratings when they tend to rate movies at a level higher than the average ratings of other users. Item popularity becomes a factor which may enhance prediction outcomes for popular movies. The model becomes adaptable due to these modifications which enable it to detect intricate patterns in the data. The system merges SVD's latent representation model with community-based detection to provide recommendations which remain true to group-specific user preferences.

## 3.2 Louvain Community-Detection Method

The core function of the Louvain algorithm within our hybrid recommendation system involves user preference cluster discovery which leads to more specific and appropriate recommendation results. The core functionality of Louvain makes it a graph clustering technique which employs modularity to separate networks into densely linked communities. The strength of network division gets quantified through Modularity $Q$ which reveals the relationship between community inner connectivity density and community interconnection density. A high value in modularity represents connected nodes within a community but shows limited connections between communities. Mathematically, modularity is defined as:

$$Q = \frac{1}{2m} \sum_{i,j} \left[ w_{ij} - \frac{k_i k_j}{2m} \right] \delta(c_i, c_j), \tag{6}$$

where:

- $w_{ij}$ represents the weight of the edge between nodes $i$ and $j$,
- $k_i$ and $k_j$ represents the degrees (sum of edge weights) of nodes $i$ and $j$,
- $m$ represents the total weight of all edges in the graph,
- $c_i$ and $c_j$ represents the communities of nodes $i$ and $j$,
- $\delta(c_i, c_j)$ is 1 if $c_i = c_j$ (same community nodes) and 0 otherwise.

Louvain proves suitable for recommendation systems because it enables detection of communities where users with corresponding taste preferences belong to the same groups. A weighted user similarity network begins the procedure through its creation

of individual user nodes and weighted edges that depict user profile similarity metrics. The calculation of similarity relies mainly on cosine similarity while following other computation metrics:

$$\text{sim}(u, v) = \frac{\sum_{i \in I_{uv}} r_{ui} r_{vi}}{\sqrt{\sum_{i \in I_{uv}} r_{ui}^2} \sqrt{\sum_{i \in I_{uv}} r_{vi}^2}}, \tag{7}$$

where:

- $r_{ui}$ represents the rating given by user $u$ to item $i$,
- $I_{uv}$ represents the set of items rated by both users $u$ and $v$.

For example, two users who frequently rate the same movies similarly will have a high similarity score. To ensure the graph remains meaningful, edges with similarity scores below a predefined threshold (e.g., 0.3) are filtered out, removing weak or noisy connections. This phase is critical to sustaining the integrity of the resulting communities and avoiding clusters formed by random or irrelevant associations.

The community detection features of Louvain provide better interpretability together with enhanced recommendation quality. Viewing the clustered data becomes important for understanding user patterns which helps develop marketing approaches and content distribution plans. The identification of users who love science fiction allows marketers to launch promotional campaigns focused on sci-fi content. Community structures help solve cold-start problems since they use initial user interactions to assign new members to existing groups which generates recommendations without needing large data collections.

Basically detected communities give practical information about user choice patterns and preferences [3, 9]. The first community groups users who watch action films separately from a community that likes watching romantic comedies. The restriction of collaborative filtering operations within specific communities enables the system to produce several performance enhancements [3, 14]. The recommendations become more precise because they originate from customers sharing identical preferences [9, 13]. The approach successfully decreases uncorrelated or inconsistent ratings thus producing better prediction outcomes [7, 13]. The SVD generates faster computational results because it analyzes smaller denser community-based matrices instead of processing the full dataset [12, 13].

The community detection features of Louvain provide better interpretability together with enhanced recommendation quality [14, 19]. Viewing the clustered data becomes important for understanding user patterns which helps develop marketing approaches and content distribution plans [3, 9]. The identification of users who love science fiction allows marketers to launch promotional campaigns focused on sci-fi content [3, 6]. Community structures help solve cold-start problems since they use initial user interactions to assign new members to existing groups which generates recommendations without needing large data collections [7, 10].

Our hybrid solution achieves strength maximization through the combination of Louvain community detection with SVD [3, 13, 14]. SVD extracts invisible components

to explain user-item interactions but Louvain enables discovery of important community structures which place these factors into relevant user communities [13, 14]. The combined method delivers scalable recommendations which additionally adapt to specific user segments' unique preferences [3, 9, 13].

### 3.3 Performance Evaluation Metrics

Our methodology includes a performance evaluation of the SVD method, which gives us a way to measure how accurate and useful the recommendation system is. SVD is very important for recommender systems, especially for collaborative filtering methods, because it finds hidden user preferences and item traits. This lets the system make very personalized suggestions based on how each user interacts with it. SVD lets the system make smart guesses even when the data is sparse or incomplete by finding hidden patterns in user-item interaction data. One of SVD's best features is that it can turn the high-dimensional user-item interaction matrix into a lower-dimensional latent space while keeping the most important information. This dimensionality reduction not only mitigates data noise but also improves computational efficiency, making the system scalable for large datasets.

When using SVD for matrix factorization (MF), the rating matrix $R$ is broken down into three latent feature matrices: $P$, $s$, and $Q$. This is shown in the equation below:

$$R \approx PsQ^T \tag{8}$$

Here:

- $R$ represents the original $m \times n$ rating matrix,
- $P$ represents $m \times m$ orthogonal matrix representing user latent features,
- $s$ represents $m \times n$ singular matrix containing importance weights,
- $Q$ represents$n \times n$ orthogonal matrix representing item latent features.

After computation, $P$ and $Q$ yield the latent feature matrices $X$ and $Y$, respectively, as follows:

$$X = P \cdot s \tag{9}$$

$$Y = Q \tag{10}$$

The matrices contain reduced-dimensional user and item representations that optimize the calculation of rating predictions. The calculation of predicted rating matrix $\tilde{R}$ involves the movement of $X$ and $Y^T$.

$$\tilde{R} = XY^T \tag{11}$$

The predictive accuracy of SVD methods gets evaluated using the variation between the real $R$ matrix and the prediction output $\tilde{R}$. The algorithm uses Root Mean Squared Error (RMSE) as a commonly adopted performance metric in recommender systems to calculate discrepancies. The prediction performance stands better

when RMSE assesses smaller average error magnitudes. The RMSE is calculated as:

$$\text{RMSE} = \sqrt{\frac{1}{P}\sum_{(i,j)}(r_{ij} - \tilde{r}_{ij})^2} \tag{12}$$

Where:

- $P$ represents the total number of predictions,
- $r_{ij}$ represents the original rating given by user $i$ to item $j$,
- $\tilde{r}_{ij}$ represents the predicted rating for user $i$ and item $j$.

The SVD model defines its precision standards through the RMSE value by reproducing genuine user-item relationship data. The model provides accurate predictions for ratings because a low RMSE value indicates successful identification of hidden data patterns. Our aim involves reducing the RMSE value through SVD integration with community detection methodologies coupled with user community group structures. The merged method supports accurate recommendation performance in addition to personalized preferences for community members thus producing specific results with various options available.

The system performance can be evaluated through different metrics like Mean Absolute Error (MAE) and precision-recall in addition to Root Mean Square Error (RMSE).

$$\text{MAE} = \frac{1}{n}\sum_{i=1}^{n}|r_i - \hat{r}_i| \tag{13}$$

Where:

- $n$ represents the total number of predictions,
- $r_i$ represents the actual rating given by the user,
- $\hat{r}_i$ represents the predicted rating generated by the recommendation system.

RMSE offers reliability as a metric because its sensitivity detects significant errors and it performs well for rating quality evaluation in low-density data situations. Such metrics enable us to perform comprehensive evaluation which proves both the effectiveness of our hybrid methodology and its better performance compared to standard approaches based on SVD.

# 4 Proposed Method

The proposed hybrid recommendation system brings together Singular Value Decomposition algorithm with Louvain community detection to embrace group-level dependencies while addressing sparsity issues and new user and diversity challenges. The first stage establishes an $R$ user-item interaction matrix as baseline data to extract user preferences as well as item characteristics. The user-item interaction matrix shows sparse distribution because user rows match items columns through the rating elements $r_{u,i}$. In explicit feedback scenarios the $r_{u,i}$ parameter stands for the user-supplied ratings ranging from 1 to 5. The rating representation value $r_{u,i}$ takes a binary format

(1 for interaction using implicit feedback) or it assigns weighted values depending on the interaction strength whether it's frequency or duration. The interaction values receive normalization treatment to eliminate biases from users who rate at uniformly high or low levels. Mean-centering adjusts user ratings by using their average score as a reference point. Implicit data collection requires converting natural interaction records into strength indicators for user-item bond measurements by adopting logarithmic methods and interaction volume formulas. Constructing a user similarity graph that depicts user relationships based on their interaction profiles becomes the subsequent process after creating the user-item interaction matrix. The user similarity graph features nodes which represent individuals and weighted edges created through the evaluation of vector interaction similarities using cosine measures. The calculation of cosine similarity between users $u$ and $v$ consists of the following formula:

$$\text{sim}(u, v) = \frac{\sum_{i \in I} R_{u,i} R_{v,i}}{\sqrt{\sum_{i \in I} R_{u,i}^2} \sqrt{\sum_{i \in I} R_{v,i}^2}} \tag{14}$$

Users' interaction values $r_{u,i}$ and $r_{v,i}$ refer to item $i$ during this calculation and $I$ signifies the set containing those items rated by both users $u$ and $v$. Two users' interaction patterns find their alignment through cosine similarity which calculates scores from -1 for complete dissimilarity to 1 for complete similarity. The graph's meaningfulness together with prevention of noisy and irrelevant connections is achieved through thresholding the cosine similarity scores. When cosine similarity between two users surpasses a specified threshold (e.g. 0.3) an edge form occurs between those users. Mathematically, this is expressed as:

$$\text{Edge}(u, v) = \begin{cases} 1, & \text{if } \text{sim}(u, v) > 0.3, \\ 0, & \text{otherwise.} \end{cases} \tag{15}$$

Such a filtering prevents weak user matches while creating a dense network structure that demonstrates grouping of like-minded users. The calculated output produces weighted undirected relationships which present users as nodes and establish weighted connections based on substantial similarities between users. The graph projection procedure changes the initial user-item interaction matrix into a structure that is compatible with Louvain and similar community detection methods. The constructed user similarity graph requires Louvain algorithm execution to determine user communities based on their preference similarities. Louvain method operates as a modularity-based technique for graph clustering which organizes networks by densely linking different communities. Modularity Q provides a measurement of network division strength by evaluating the density ratio of community internal connections to external connections. The high value of modularity reveals that distinct communities contain nodes strongly connected to each other yet have few external connections between communities. The first phase of the algorithm consists in successive assessments of modularity improvement by relocating nodes between adjacent communities. The subsequent phase combines communities into single nodes until no further increases in modularity

are possible. The step culminates in user clusters that each contains people with equivalent preferences. These clusters form the basis for personalized recommendations, as they allow us to restrict collaborative filtering to within-community interactions, reducing noise and improving accuracy. Once the communities are identified, the next step is to apply Singular Value Decomposition (SVD) within each community to unveil implicit elements that explain user-item interactions. For each community, a submatrix of the original user-item collaboration matrix $R$ is extracted, containing only the interactions of users within that community. This submatrix is denoted as $R_c$, where $c$ represents the community index. SVD is then performed on $R_c$ to decompose it into three components:

$$R_c \approx P_c \Sigma_c Q_c^T \tag{16}$$

Here, $P_c$ is an $mc \times k$ matrix representing user latent features, $\Sigma_c$ is a $k \times k$ diagonal matrix containing singular values, and $Q_c$ is an $nc \times k$ matrix representing item latent features. The decomposition reduces the high-dimensional user-item interaction matrix into a lower-dimensional latent space, capturing hidden relationships between users and items. During training, user and item latent factor vectors are iteratively updated using stochastic gradient descent (SGD), minimizing the squared error between predicted and actual ratings while incorporating regularization terms to prevent overfitting. The prediction formula incorporates biases such as global average ratings, user-specific tendencies, and item popularity, ensuring flexibility and interpretability. For example, the predicted rating $\hat{r}_{u,i}$ for user $u$ and item $i$ is computed as:

$$\hat{r}_{u,i} = \mu + b_u + b_i + p_u^T q_i \tag{17}$$

Where $\mu$ is the global average rating, $bu$ is the user-specific bias, $bi$ is the item-specific bias, $pu$ is the latent factor vector for user u, and $qi$ is the latent factor vector for item i. By applying SVD within communities, the system ensures that recommendations are personalized to the specific tastes of user groups, reducing noise from unrelated or outlier ratings and improving computational efficiency.

The SVD model delivers predicted ratings of unrated items to each community before the recommendation system produces top-k rated items for users. The Louvain algorithm identifies user communities before applying results for individual user prediction. The SVD model predicts ratings for every item which the user has not rated within its specific community. The items receive descending ratings based on their predicted scores and the top-k rated items with highest scores become recommended options for the user. The recommendation system delivers both personalized user-specific results and popular items from their community therefore it provides enhanced relevance alongside diverse matching content. The value of k in the recommendation system enables users to specify their desired number of suggestions and interface constraints allowing them to receive personalized recommendations.

A system evaluation consists of two measuring metrics that assess predicted rating accuracy through Root Mean Squared Error (RMSE) (Eqn.11) and Mean Absolute Error (MAE) (Eqn.12).

# 5 Results and Discussion

A thorough evaluation of the proposed recommendation system occurred through analysis of the widely used Netflix Prize dataset in recommendation systems research. The dataset more than 24 million ratings from about 470758 users who have rated 4499 movies at each level of 1 to 5 within an integer rating system.

**Table 1** Dataset statistics from the Netflix Prize (Kaggle).

| Dataset | Users | Movies | Total Ratings | Rating Range | Sparsity |
|---|---|---|---|---|---|
| Netflix Prize (Kaggle) | 470758 | 4499 | 24053764 | 1 to 5 | 0.9886 |

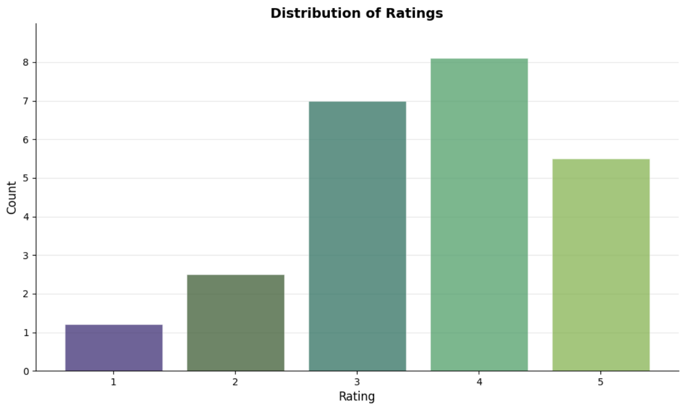


**Fig. 1** Distribution of ratings for movies across the dataset.

Root Mean Squared Error (RMSE) and Mean Absolute Error (MAE) metrics were used to evaluate our hybrid system, which used Singular Value Decomposition as its core algorithm. The results were measured using three different splits from the dataset to establish reliable findings. A 100-user and 50-movie randomized selection was implemented to investigate user preference habits through an experimental analysis. The experimental goal targeted the visible detection of rating data communities together with rating pattern analysis between different user collections. A user graph consisted of 100 nodes and 631 edges after our analysis used a 0.3 similarity threshold to determine edge connections. Louvain community detection algorithms revealed six separate clusters inside the data set which contained between 11 and 25 user members:

- Community 0: 25 users
- Community 1: 17 users
- Community 2: 11 users
- Community 3: 12 users

- Community 4: 13 users
- Community 5: 22 users

Figure 2 illustrates the network visualization of these communities, demonstrating the clustering of users based on their rating behaviors. Figure 3 displays the user distribution within each of the communities that were identified. The visualization highlights how users with similar preferences naturally group together in the recommendation space.

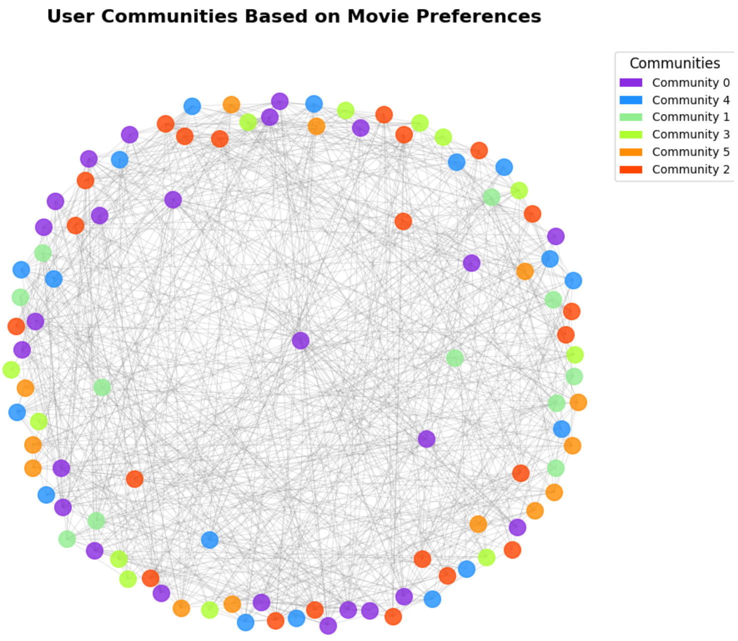


**Fig. 2** Network visualization of six detected user communities based on movie rating patterns.

We analyzed the average ratings for top movies across different communities to identify community-specific preferences. Figure 4 presents a heatmap of the community-movie matrix for the 15 highest-rated movies, revealing distinct preference patterns among the detected communities. This visualization demonstrates how certain movies are...

Table 2 presents the experimental outcomes for SVD and includes RMSE and MAE ratings per fold with mean and standard deviation calculation. The analysis results across three folds demonstrated that RMSE reached values of 0.9957 and 0.9943 and 0.9998 for a mean RMSE of 0.9966 alongside a standard deviation of 0.0023. The obtained mean of MAE obtained from three data splits was 0.7968 while its standard deviation reached 0.0007. The SVD algorithm demonstrated stable performance

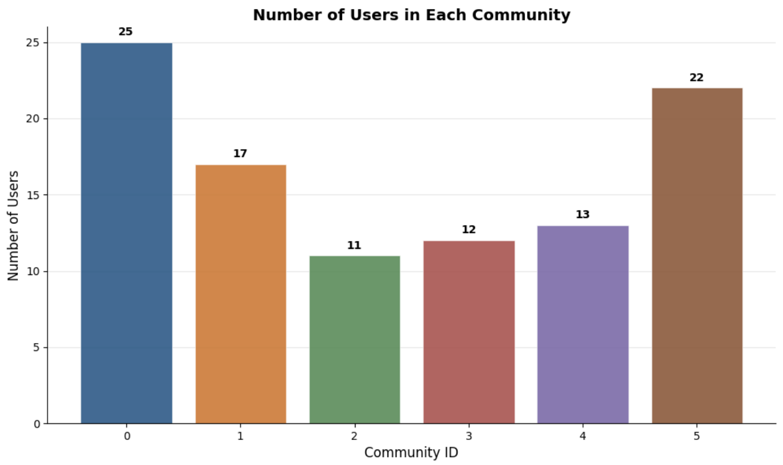


**Fig. 3** Distribution of users in each of the detected communities.

**Table 2** Results for Singular Value Decomposition on test set.

| **Metric** | **Fold 1** | **Fold 2** | **Fold 3** | **Mean** | **Std. Deviation** |
|---|---|---|---|---|---|
| RMSE (test set) | 0.9957 | 0.9943 | 0.9998 | 0.9966 | 0.0023 |
| MAE (test set) | 0.7970 | 0.7959 | 0.7975 | 0.7968 | 0.0007 |
| Fit Time (seconds) | 1.62 | 2.53 | 4.37 | 2.84 | 1.14 |
| Test Time (seconds) | 0.30 | 0.41 | 0.66 | 0.46 | 0.15 |

through the splits of the dataset as shown by its low variation in RMSE and MAE values. The model's stability is seen in this figure 1 which shows the visual representation of RMSE and MAE values from each fold.

The measurement of algorithm efficiency through computational speed included fit time and test time assessments for all fold simulations. The behavior of the fitting procedure involved times of 1.62 seconds, 2.53 seconds and 4.37 seconds which produced an average fit time of 2.84 seconds with standard deviation of 1.14 seconds. Each record took a mean duration of 0.46 seconds to execute while the respective standard deviation measured 0.15 seconds. The time periods under observation were 0.30, 0.41, and 0.66 seconds. The Netflix Prize-scale dataset requires the SVD algorithm to operate efficiently given the outcomes presented here. You will find complete data on fit and test duration specifics for each fold of the experiment in Table 2.

The independent SVD algorithm's performance will potentially receive additional enhancement when it gets merged with Louvain community detection for better recommendation accuracy and personalization outcomes. As a result of user clustering the hybrid model optimizes predictive accuracy by concentrating the analysis on connected clusters that minimize rating noise from unrelated entries. The computational speed improves and prediction precision strengthens because SVD operates on individual community submatrices which are denser and smaller in size. The research team

aims to develop the framework by adding features involving temporal data analysis and implicit feedback evaluation procedures to improve recommendation systems.

A 100-user and 50-movie randomized selection was implemented to investigate user preference habits through an experimental analysis. The experimental goal targeted the visible detection of rating data communities together with rating pattern analysis between different user collections. A user graph consisted of 100 nodes and 631 edges after our analysis used a 0.3 similarity threshold to determine edge connections. Louvain community detection algorithms revealed six separate clusters inside the data set which contained between 11 and 25 user members:

- Community 0: 25 users
- Community 1: 17 users
- Community 2: 11 users
- Community 3: 12 users
- Community 4: 13 users
- Community 5: 22 users

Figure 2 illustrates the network visualization of these communities, demonstrating the clustering of users based on their rating behaviors. The user distribution in each of these identified communities is displayed in Figure 3. The visualization highlights how users with similar preferences naturally group together in the recommendation space.

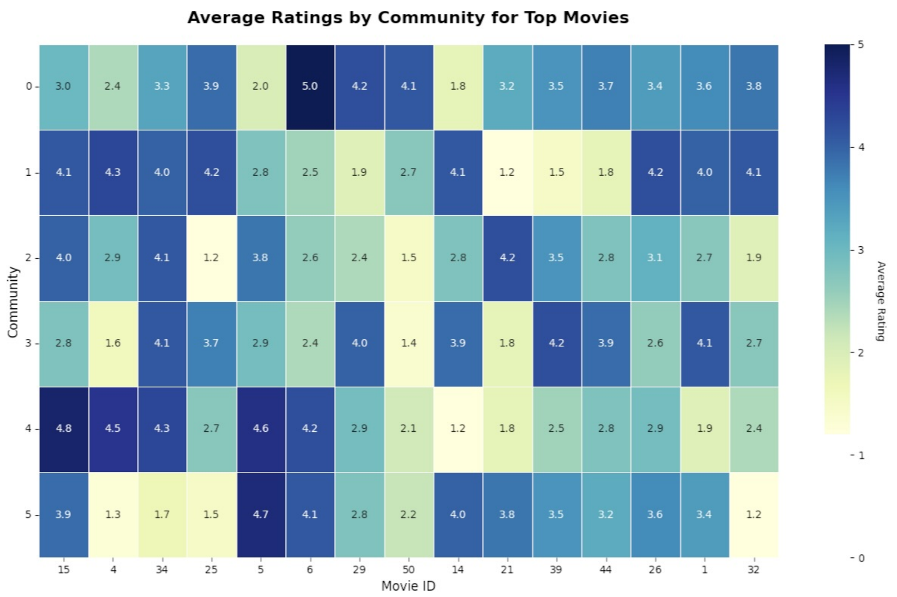


**Fig. 4** Heatmap of the community-movie matrix for the 15 highest-rated movies

We analyzed the average ratings for top movies across different communities to identify community-specific preferences. Figure 4 presents a heatmap of the

community-movie matrix for the 15 highest-rated movies. This visualization demonstrates how certain movies are consistently rated higher within specific communities, supporting the effectiveness of community-based recommendations.

In conclusion, the outcomes of the experiment demonstrate that the SVD algorithm functions effectively as the foundation for the suggested hybrid recommendation system. Thus, the model is very good at making predictions and is also very fast at doing calculations, with a mean RMSE of 0.9966 and a mean MAE of 0.7968. These results set the stage for adding community detection methods to the system in the future to make it even more accurate and personalized.

## 6 Conclusion and Future Work

The Louvain community detection method in conjunction with SVD has brought forth an optimal strategic solution which transforms the landscape of recommendation systems [3, 9, 13]. The harmonious combination provides scalable accurate and elevated personal recommendation results at the same time [13, 14]. SVD processing enabled our system to take advantage of user-item patterns in order to create a selection of targeted and relevant recommendations [7, 12, 13]. The Louvain community detection method functioned as an essential component which identified important clusters among users according to their shared preferences [3, 14, 19].

The application of this approach received experimental verification using both 100 users and 50 movies in a sample dataset. Louvain algorithm generated six distinct user clusters with membership ranging between 11 and 25 users which proves its capability to identify real preference clusters from the population [14, 19, 20]. The use of network graphs and heatmaps allowed visualizing user communities to reveal important collective behavioral patterns that standard recommendation systems fail to detect [9, 10, 19]. Visual representations reveal how distinct rating behaviors exist between different community groups meaning that it proves the necessity of creating content recommendations that cater to specific groups [7, 14].

Our method demonstrates its practical use by providing tailor-made suggestions to community members which proves both effective recommendations and high computation speed [3, 13, 19]. Through the use of community structures our system delivered more suitable recommendations which correspond to the shared preferences of users belonging to similar communities [9, 13, 14].

The recommendation system has evolved via fine adjustments to create individualized and more satisfactory experiences for users [3, 7, 10]. The use of SVD and Louvain community detection techniques yield a complete solution for developing robust recommendation systems which scale up effectively [3, 13, 14]. The solution yields faster calculations and better recommendation predictions which effectively helps modern recommendation systems manage their requirements [7, 13, 14].

Future investigations should focus on applying SVD with Louvain for enhancing cross-domain recommendation systems [9, 11, 14]. The approach would employ combined information across several domains to present users with broader and various recommendations that cater to specific user interests and preferences [3, 7, 9]. Enhancements of our methodology could be achieved by implementing temporal analysis which

would detect changes in community patterns and user preferences throughout time [11, 17, 20].

Our work should also focus on perfecting the execution of this combined method when implementing it for large-scale recommendation systems [14, 19, 20]. Efforts to develop interpretable recommendation explanations through community membership analysis show promising results for building user trust and satisfaction towards the system [8, 13, 18]. The development of interpretable recommendation explanations based on community membership represents another promising direction [8, 14].

# 7 References

# References


[1] Mohammadi M, Fazlali M, Hosseinzadeh M (2022) Parallel Louvain Community Detection Algorithm Based on Dynamic Thread Assignment on Graphic Processing Unit. Journal of Electrical and Computer Engineering Innovations (JECEI) 10(1):75–88. https://doi.org/10.22061/JECEI.2021.7771.432

[2] C. Gao, Y. Zheng, N. Li, et al., "A Survey of Graph Neural Networks for Recommender Systems: Challenges, Methods, and Directions," *arXiv preprint arXiv:2109.12843*, 2022. [Online]. Available: https://arxiv.org/abs/2109.12843

[3] F. Gasparetti, G. Sansonetti, and A. Micarelli, "Community Detection in Social Recommender Systems: A Survey," *Applied Intelligence*, vol. 51, no. 6, pp. 3975–3995, 2021, doi:10.1007/s10489-020-02148-7. [Online]. Available: https://doi.org/10.1007/s10489-020-02148-7

[4] M. Li, L. Zhao, Y. Ren, et al., "Graph Neural Network Recommendation Algorithm Based on Improved Dual-Tower Model," *Gels*, vol. 11, no. 2, p. 141, 2024, doi:10.3390/gels11020141. [Online]. Available: https://doi.org/10.3390/gels11020141

[5] J. Zhang, J. Fei, X. Song, and J. Feng, "An Improved Louvain Algorithm for Community Detection," *arXiv preprint arXiv:2110.00891*, 2021. [Online]. Available: https://arxiv.org/abs/2110.00891

[6] Y. Zhao, C. Zhou, J. Cao, et al., "Building a Movie Recommendation System Using SVD Algorithm," *Scientific Reports*, vol. 14, p. 3853, 2024, doi:10.1038/s41598-024-54303-6. [Online]. Available: https://doi.org/10.1038/s41598-024-54303-6

[7] L. Chen and W. Zhang, "Movie Recommendation System Based on Collaborative Filtering and Matrix Factorization," *Journal of Emerging Technologies and Innovative Research (JETIR)*, 2023. [Online]. Available: https://www.jetir.org/papers/JETIR2306709.pdf

[8] Z. Shokrzadeh, M.-R. Feizi-Derakhshi, M.-A. Balafar, and J. Mohasefi, “Graph-Based Recommendation System Enhanced by Community Detection,” *Wireless Communications and Mobile Computing*, vol. 2023, Article ID 5073769, 2023, doi:10.1155/2023/5073769. [Online]. Available: https://doi.org/10.1155/2023/5073769

[9] S. Wu, F. Sun, W. Zhang, et al., “Graph Neural Networks in Recommender Systems: A Survey,” *ACM Computing Surveys*, vol. 55, no. 7, pp. 1–37, 2022, doi:10.1145/3535101. [Online]. Available: https://doi.org/10.1145/3535101

[10] C. Choudhary, I. Singh, and M. Kumar, “Community Detection Algorithms for Recommendation Systems: Techniques and Metrics,” *Computing*, vol. 105, pp. 1–24, 2022, doi:10.1007/s00607-022-01131-z. [Online]. Available: https://doi.org/10.1007/s00607-022-01131-z

[11] S. Funk, “Netflix Update: Try This at Home,” 2006. [Online]. Available: http://sifter.org/~simon/journal/20061211.html (accessed Nov. 21, 2024)

[12] Y. Koren, R. Bell, and C. Volinsky, “Matrix Factorization Techniques for Recommender Systems,” *Computer*, vol. 42, no. 8, pp. 30–37, 2009, doi:10.1109/MC.2009.263. [Online]. Available: https://doi.org/10.1109/MC.2009.263

[13] V. D. Blondel, J.-L. Guillaume, R. Lambiotte, and E. Lefebvre, “Fast Unfolding of Communities in Large Networks,” *Journal of Statistical Mechanics: Theory and Experiment*, vol. 2008, no. 10, p. P10008, 2008, doi:10.1088/1742-5468/2008/10/P10008. [Online]. Available: https://doi.org/10.1088/1742-5468/2008/10/P10008

[14] Hamilton WL, Ying R, Leskovec J (2017) Inductive Representation Learning on Large Graphs. In: Advances in Neural Information Processing Systems (NeurIPS) 30. arXiv preprint arXiv:1706.02216

[15] van den Berg R, Kipf TN, Welling M (2017) Graph Convolutional Matrix Completion. In: Proceedings of the 24th ACM SIGKDD International Conference on Knowledge Discovery & Data Mining. arXiv preprint arXiv:1706.02263

[16] L. Xia, C. Huang, Y. Xu, P. Dai, and L. Bo, ”Multi-Behavior Graph Neural Networks for Recommender System,” arXiv preprint arXiv:2302.08678, Feb. 2023.

[17] H. Zhu, V. Kapoor, and P. Sharma, ”Reviewing Developments of Graph Convolutional Network Techniques for Recommendation Systems,” arXiv preprint arXiv:2311.06323, Nov. 2023.

[18] V. D. Blondel, J.-L. Guillaume, R. Lambiotte, and E. Lefebvre, ”Fast Unfolding of Communities in Large Networks,” Journal of Statistical Mechanics: Theory

and Experiment, vol. 2008, no. 10, p. P10008, Oct. 2008, doi: 10.1088/1742-5468/2008/10/P10008.

[19] L. Yang, Y. Wang, and J. Tang, "Graph Neural Networks in Recommender Systems: A Survey," arXiv preprint arXiv:2011.02260, Nov. 2020.

[20] S. Wu, F. Sun, W. Zhang, Y. Zhu, and B. Cui, "Graph Neural Networks in Recommender Systems: A Survey," ACM Comput. Surv., vol. 55, no. 7, pp. 1–37, 2022, doi: 10.1145/3535101.

[21] K. Sharma, Y.-C. Lee, S. Nambi, and Y. Park, "A Survey of Graph Neural Networks for Social Recommender Systems," *ACM Computing Surveys*, 2024. [Online]. Available: https://arxiv.org/abs/2212.04481

[22] S. Wu, W. Zhang, F. Sun, and B. Cui, "Graph Neural Networks in Recommender Systems: A Survey," *arXiv preprint arXiv:2011.02260*, 2020. [Online]. Available: https://arxiv.org/abs/2011.02260

[23] C. Gao, Y. Zheng, N. Li, et al., "A Survey of Graph Neural Networks for Recommender Systems: Challenges, Methods, and Directions," *arXiv preprint arXiv:2109.12843*, 2021. [Online]. Available: https://arxiv.org/abs/2109.12843

[24] X. He, "Graph Neural Networks in Recommender Systems," *Applied Computational Engineering*, vol. 79, pp. 234–240, 2024, doi:10.54254/2755-2721/79/20241646. [Online]. Available: https://doi.org/10.54254/2755-2721/79/20241646

[25] T. Duricic, D. Kowald, E. Lacic, and E. Lex, "Beyond-Accuracy: A Review on Diversity, Serendipity and Fairness in Recommender Systems Based on Graph Neural Networks," *arXiv preprint arXiv:2310.02294*, 2023. [Online]. Available: https://arxiv.org/abs/2310.02294

[26] H. Zhu, V. Kapoor, and P. Sharma, "Reviewing Developments of Graph Convolutional Network Techniques for Recommendation Systems," *arXiv preprint arXiv:2311.06323*, 2023. [Online]. Available: https://arxiv.org/abs/2311.06323

[27] V. D. Blondel, J.-L. Guillaume, R. Lambiotte, and E. Lefebvre, "Fast Unfolding of Communities in Large Networks," *Journal of Statistical Mechanics: Theory and Experiment*, vol. 2008, no. 10, p. P10008, Oct. 2008, doi:10.1088/1742-5468/2008/10/P10008. [Online]. Available: https://doi.org/10.1088/1742-5468/2008/10/P10008

[28] Y. Koren, R. Bell, and C. Volinsky, "Matrix Factorization Techniques for Recommender Systems," *Computer*, vol. 42, no. 8, pp. 30–37, Aug. 2009, doi:10.1109/MC.2009.263. [Online]. Available: https://doi.org/10.1109/MC.2009.263

[29] S. L. Jothilakshmi and R. Bharathi, "Survey on Collaborative Filtering Technique for Recommender System Using Deep Learning," in *Computer Vision and Machine Intelligence Paradigms for SDGs*, Springer, 2023, pp. 217–225.

[30] V. Lakshmi Chetana and H. Seetha, "A Study on Transparent Recommender Systems," in *Explainable, Interpretable, and Transparent AI Systems*, CRC Press–Taylor & Francis Group, 2024, ISBN 9781003442509. [Online]. Available: https://link.springer.com/chapter/10.1007/978-981-19-7169-3_20

[31] S. T. Soman, S. V. J. Soumya, and K. P. Soman, "Singular Value Decomposition: A Classroom Approach," *International Journal of Recent Trends in Engineering*, vol. 2, no. 1, 2009.
