# Supplementary material for: Enhancing Recommendation Systems through SVD-based collaborative filtering and community detection
Source: PLoS One. 2026 Apr 13;21(4):e0346579. doi: 10.1371/journal.pone.0346579 (PMC13075704; doi:10.1371/journal.pone.0346579)
Supplement: Attachment — Submitted filename: Response to Reviewers.docx [file pone.0346579.s002.docx]

**Manuscript Title:** Enhancing Recommendation Systems through SVD-Based Collaborative Filtering and Community Detection

**Authors:** Keerthika T, Ignisha Rajathi G, Vedhapriyavadhana Rajamani, Surya Santhosh Kumar, Karthigai Selvam, Aiyyappan M R, L S Thoshi Babu

**Dear Academic Editor and Reviewers,**

We would like to thank you for the opportunity to revise our manuscript. We appreciate the thoughtful and constructive comments provided by the reviewers, which have significantly helped us improve the quality, clarity, and technical rigor of our work.

We have addressed each comment point-by-point below and have highlighted the corresponding changes in the revised manuscript. Major revisions include the addition of a comparative analysis against GNNs, a rigorous complexity analysis, hyperparameter tuning details, and a complete restructuring of the text into the passive voice as requested.

### Response to Reviewer #1

Comment 1-4 & 6: The reviewer noted that the article aligns well with the journal's scope, is organized, and clearly presents the methodology.

Response: We thank Reviewer #1 for their encouraging comments and for recognizing the alignment of our work with the journal's scope. We appreciate the positive feedback regarding the clarity of our presentation.

Comment 5: The authors concluded that these metrics demonstrate high performance compared to isolated SVD implementations and offer significantly better interpretability than GNN-based approaches.

Response: We appreciate this observation. To substantiate this conclusion with concrete evidence, we have added a new "Comparative Analysis and Discussion" subsection in the Results section.

- **Table 3** has been added to explicitly benchmark our model’s RMSE (0.9966) against isolated SVD (typically ~1.05 on sparse data) and GNN-based approaches (typically ~0.90-0.95).
- We have expanded the discussion to highlight that while GNNs offer a slight accuracy edge, our hybrid method offers superior **interpretability** through community structures, validating the trade-off claimed in our conclusion.

### Response to Reviewer #2

Comment 1: The abstract makes strong claims... but does not clearly explain the novelty... Adding a clearer statement of the methodological contribution would strengthen the abstract.

Response: We have rewritten the Abstract to explicitly define the "sparsity-interpretability trade-off" as the core problem. We clarified that our novelty lies in using Louvain modularity not just for regularization, but as a pre-processing step to create dense, homogenous sub-matrices for the SVD, thereby reducing noise.

Comment 2: The description of experimental results lacks context—no baseline RMSE/MAE values...

Response: We have added a "Comparative Analysis" subsection in the Results section. This includes Table 3, which provides standard literature benchmarks for Standard User-CF, Basic SVD, and Neural CF (Deep Learning), allowing readers to contextualize our RMSE of 0.9966.

Comment 3: The introduction should clearly conclude with a distinct section highlighting the novel contributions.

Response: We have added a bulleted "Contributions" subsection at the end of the Introduction to explicitly list the three key contributions of this study: the novel hybrid architecture, enhanced interpretability, and scalable performance.

Comment 4: Add a para that mentions briefly what each next section contains.

Response: We have added an "Organization of the Paper" subsection at the end of the Introduction that outlines the structure of the subsequent sections.

Comment 5: The literature review should benefit from more explorations of previous studies.

Response: We have expanded the Related Work section to include a discussion on recent studies involving side-information and lightweight community detection methods, positioning our work within this broader context.

Comment 6: The discussion section needs to be expanded to more thoroughly analyze the results.

Response: We have expanded the Results and Discussion section to explain why the proposed method performs well. Specifically, we discuss how the "divide-and-conquer" strategy isolates noise within specific communities, leading to more precise latent feature extraction.

Comment 7: The first paragraph of the conclusion should succinctly summarize the contributions of the study in past tense.

Response: We have rewritten the first paragraph of the Conclusion in the past tense to summarize exactly what was done and what was achieved.

Comment 8: The second paragraph of the conclusion should provide clear and actionable future recommendations.

Response: We have revised the future work paragraph to be specific and actionable, suggesting the incorporation of temporal dynamics and testing on distributed computing frameworks.

Comment 9: Equations are not properly cited.

Response: We have added the appropriate citations (e.g., [Soman et al.], [Blondel et al.]) immediately preceding the mathematical formulations for SVD and Modularity in the Methodology section.

Comment 10: Manuscript is highly low on visualizations.

Response: To address this, we have added Figure 6, a methodological flowchart that visually describes the step-by-step architecture of the proposed system. We have also added Figure 7, a bar chart comparing our RMSE against baselines.

### Response to Reviewer #3

Comment 1: The introduction can include more recent state of the art works.

Response: We have updated the Introduction to reference recent trends, including Transformer-based models and Large Language Models (LLMs) for sequential recommendation, to provide a more current state-of-the-art context.

Comment 2: Please specify the novelty and contributions of the work.

Response: As noted in our response to Reviewer #2, we have added a specific "Contributions" subsection to the Introduction.

Comment 3: The assumptions... along with possible identifiability SVD conditions should be mentioned.

Response: We have added a new subsection titled "Assumptions and Theoretical Constraints" in the Methodology section. This explicitly outlines our assumptions regarding the Low-Rank nature of the matrix, Homophily, and SVD Identifiability.

Comment 4 & 6: It is not clear how to choose the learning rate and regularization parameter... Simulations should show the impact.

Response: We have added a "Hyperparameter Tuning" subsection in the Proposed Method section. This details our Grid Search strategy and reports the specific values selected ($\alpha = 0.005$, $\lambda = 0.02$) and the observed effects of varying these parameters.

Comment 5: It is not clear how diversity is treated.

Response: We have added a "Discussion on Diversity and Personalization" in the Results section. We explain that while our primary metric is RMSE, the community-based approach inherently improves diversity by mitigating popularity bias and surfacing niche items relevant to specific user groups.

Comment 7: The authors should conduct a rigorous computational complexity analysis.

Response: We have added a "Computational Complexity Analysis" subsection in the Methodology. We use Big-O notation to demonstrate that our approach (O(N log N) for clustering + Local SVD) is computationally more efficient than standard GNN-based methods (O(L . |R| + L . N . d^2)).

Comment 8: The work should compare with more state of the art methods.

Response: We have addressed this via Table 3 and the new Figure 7, which compare our method against GNN-based benchmarks (like NGCF) and standard SVD.

Comment 9: Please fix the text size of the figure, for example Fig. 1 has very small text.

Response: We have revised Figure 1 to ensure that the axis labels and text are legible and of appropriate size for publication.

### Response to Reviewer #4

Comment 1: Avoid the word “we”, “our”... Write in passive voice.

Response: We have meticulously revised the entire manuscript (Abstract, Introduction, Conclusion, and Methodology) to replace active voice (e.g., "We propose") with passive voice (e.g., "A method is proposed").

Comment 2: The citations in the Introduction have referred randomly... Rearrange the reference list.

Response: We have completely reordered the Bibliography and citations throughout the text. References now appear sequentially, starting with [1], [2] in the Introduction.

Comment 3: Write concluding remarks at the end of the related work about what new thing has been focused on...

Response: We have added a subsection "Concluding Remarks on Research Gap" at the end of Related Work to clearly articulate the gap our study fills regarding the trade-off between sparsity and interpretability.

Comment 4: Include a flowchart at the beginning of section 4.

Response: We have inserted Figure 6 (Architectural Workflow) at the beginning of the Proposed Method section (Section 4) to provide an immediate visual overview of the system design.

Comment 5: Figure 1 is not mentioned in the text.

Response: We have added an explicit reference to Figure 1 in the Results section, using it to discuss the long-tail distribution of the dataset ratings.

Comment 6: Elaborate Figure 2, 3 and 4 in more details.

Response: We have added detailed descriptive text in the Results section interpreting specific features of these figures (e.g., interpreting the connectivity density in the network visualization and the user distribution across clusters).

Comment 7 & 9: Results section needs improvement... include isolated SVD and GNN methods results in a single plot.

Response: We have added Figure 7, a comparative bar chart that visually displays the RMSE of Isolated SVD, GNN, and the Proposed Hybrid method side-by-side, validating our performance claims.

Comment 8: Include a comparative table.

Response: We have included Table 3 in the Results section, which compares Methodology, RMSE, Sparsity Handling, and Interpretability across the different approaches.

Comment 10: Specify some limitations of the proposed method in the conclusion.

Response: We have added a paragraph to the Conclusion explicitly stating the limitations of our method, specifically regarding the "cold-start" issue for new users and the computational cost of periodic re-clustering.

Comment 11: Check minor typos and grammatical errors.

Response: We have proofread the manuscript and corrected typos and grammatical errors to ensure a formal academic tone.
