# Supplementary material for: Enhancing Recommendation Systems through SVD-based collaborative filtering and community detection
Source: PLoS One. 2026 Apr 13;21(4):e0346579. doi: 10.1371/journal.pone.0346579 (PMC13075704; doi:10.1371/journal.pone.0346579)
Supplement: Attachment — Submitted filename: Response to Reviewers-2.docx [file pone.0346579.s003.docx]

**Response to Reviewers**

**Title: Enhancing Recommendation Systems through SVD-Based Collaborative Filtering and Community Detection**

Dear Editor and Reviewers,

We sincerely thank the Editor and all Reviewers for their constructive feedback and positive assessments of our manuscript. The comments have been very helpful in improving the clarity, rigor, and presentation of our work. We have carefully addressed every point raised, with changes highlighted in the "Revised Manuscript with Track Changes" file.

As per the recent mail, the following has been carefully updated in the manuscript.

**Data Availability:**

The dataset used in this study is the publicly available Netflix Prize dataset, which contains anonymized movie rating interactions between users and movies and is widely used for benchmarking recommender systems. The dataset includes millions of user–item ratings collected during the Netflix Prize competition and is designed for research in collaborative filtering and recommendation algorithms.

The dataset can be accessed from the Kaggle repository at:

{https://www.kaggle.com/datasets/netflix-inc/netflix-prize-data}.

The dataset from the original is reproduced in the following dataset

doi:10.6084/m9.figshare.31428857

Thank you again for your time and consideration. We look forward to your decision.

Sincerely, Ignisha Rajathi G (Corresponding Author) Manipal Institute of Technology Bengaluru, Manipal Academy of Higher Education Email: [ignisha.rajathi@manipal.edu](mailto:ignisha.rajathi@manipal.edu)

On behalf of all authors: Keerthika T, Vedhapriyavadhana Rajamani, Surya Santhosh Kumar, Karthigai Selvam, Aiyyappan M R, L S Thoshi Babu.
